# Biphasic Calcium Phosphate and Activated Carbon Microparticles in a Plasma Clot for Bone Reconstruction and In Situ Drug Delivery: A Feasibility Study

**DOI:** 10.3390/ma17081749

**Published:** 2024-04-11

**Authors:** Samah Rekima, Nadine Gautier, Sylvie Bonnamy, Nathalie Rochet, Florian Olivier

**Affiliations:** 1 INSERM, CNRS, iBV, Université Côte d’Azur, 06107 Nice, France; samah.rekima@univ-cotedazur.fr (S.R.); nadine.gautier@univ-cotedazur.fr (N.G.); nathalie.rochet@univ-cotedazur.fr (N.R.); 2 CNRS, Université d’Orléans, ICMN UMR 7374, 45071 Orléans, France; sylvie.bonnamy@cnrs-orleans.fr

**Keywords:** activated carbon, biphasic calcium phosphate, plasma clot, tacrolimus adsorption/desorption, drug delivery system, bone filling

## Abstract

The development of bone-filling biomaterials capable of delivering in situ bone growth promoters or therapeutic agents is a key area of research. We previously developed a biomaterial constituting biphasic calcium phosphate (BCP) microparticles embedded in an autologous blood or plasma clot, which induced bone-like tissue formation in ectopic sites and mature bone formation in orthotopic sites, in small and large animals. More recently, we showed that activated carbon (AC) fiber cloth is a biocompatible material that can be used, due to its multiscale porosity, as therapeutic drug delivery system. The present work aimed first to assess the feasibility of preparing calibrated AC microparticles, and second to investigate the properties of a BCP/AC microparticle combination embedded in a plasma clot. We show here, for the first time, after subcutaneous (SC) implantation in mice, that the addition of AC microparticles to a BCP/plasma clot does not impair bone-like tissue formation and has a beneficial effect on the vascularization of the newly formed tissue. Our results also confirm, in this SC model, the ability of AC in particle form to adsorb and deliver large molecules at an implantation site. Altogether, these results demonstrate the feasibility of using this BCP/AC/plasma clot composite for bone reconstruction and drug delivery.

## 1. Introduction

Bone defects are frequently associated with other pathological situations such as infection or malignancy. In such cases, the ideal treatment should be to fill the defect with a biomaterial that can induce bone reconstruction and control the associated pathology [1,2]. Synthetic biomaterials appear to be the most suitable grafting materials for combining these properties. Calcium phosphates (CaP), such as hydroxyapatite (HA), alpha- or beta-tricalcium phosphate (α- or β-TCP), biphasic calcium phosphate (BCP), and calcium-deficient apatites (CDA), are commonly used as bone-filling biomaterials in bone reconstructive surgery. They are available in various forms such as granules, dense or porous ceramic pieces, cements and coatings [3,4,5,6,7,8].

We have previously shown that calibrated BCP microparticles embedded in a blood or plasma clot constitute a new, easy-to-handle bone substitute that can reconstruct bone defects of various shapes and sizes [9,10]. This BCP/blood composite, named “BRB”, is able to induce immature woven bone formation after subcutaneous (SC) implantation in mice and to repair critical-sized femoral defects in rats [9]. Our experiments revealed that particles sized between 80 and 200 µm were optimal for bone colonization and that maximal particle density into the clot was crucial for bone repair. More recently, we have shown that this biomaterial is able to induce the reconstruction of critical interruptive long bone defects in dogs and that the newly formed bone has high regenerative potential [10]. 

Activated carbons (ACs) have long been used for the adsorption/desorption of diverse molecules, among them medical molecules, thanks to their physico-chemical characteristics (e.g., surface area, level of microporosity, and surface active sites) [11,12,13,14]. Recently, carbon-based nanomaterials have attracted particular attention in bone reconstruction [15,16,17]. The main limitation of these carbon nanomaterials is their size, which is generally less than 200 nm, with the risk of being eliminated by the human immune system or the target organ [18,19,20]. We and others have shown that ACs, under the form of carbon fibers, are interesting materials for bone reconstruction [21,22,23,24,25,26]. In pilot studies performed in a rat model of large bone cortical defects, we have shown that activated carbon fiber cloth is highly biocompatible and fully osteointegrated after 6 months post-surgery [27]. Furthermore, we also demonstrated that AC cloth can be used as an efficient drug delivery system [28].

The present study was performed to evaluate two critical points: firstly, the feasibility to prepare calibrated AC microparticles and to incorporate them, alone or combined with BCP microparticles, into a plasma clot; secondly, the feasibility to use the porosity of the AC microparticles to release, in situ, a selected drug. To answer these questions, we used a well-known SC implantation model in mice [29] described previously [30,31].

## 2. Materials and Methods

### 2.1. Biphasic Calcium Phosphate (BCP) Particles

Biphasic calcium phosphate (BCP) particles, constituting 60% hydroxyapatite [HA, Ca_10_(PO_4_)_6_(OH)_2_] and 40% β-tricalcium phosphate [β-TCP, β-Ca_3_(PO_4_)_2_], were provided by Graftys SA (Aix-en-Provence, France). This CaP ceramic was obtained by sintering (1050 °C for 5 h) calcium-deficient apatite presenting a Ca/P ratio of 1.6 and prepared via an aqueous alkaline hydrolysis method. BCP microparticles measuring 80–200 µm were prepared via crushing and wet sieving, then dried and made endotoxin-free via heating at 250 °C for 1 h. Microparticles were dried and sterilized via heating at 180 °C for 2 h before use.

### 2.2. Activated Carbon (AC) Particles

The activated carbon (AC) particles, named L27, were provided by Jacobi Carbons^®^ (Vierzon, France). They were sieved to obtain 80–160 µm or 160–250 µm microparticles, dried at 80 °C for 24 h, and then sterilized via heating at 180 °C for 2 h before use.

### 2.3. Drug Adsorption into AC Particles

Adsorption was carried out in a 1 mL syringe by immersing particles for 24 h at room temperature in 625 µL of the following solutions: either ethanol (EtOH 96%, used as vehicle), NaCl 0.9% (used as control), or Tacrolimus (TA, FK-506 monohydrate, Merk, Lyon, France) solution in 96% EtOH. Two different concentrations of TA were tested, namely TA_1_ = 800 ppm and TA_2_ = 160 ppm. The particles constituted either AC alone (10 mg of 80-160 µm-sized) or BCP/AC (25 mg/5 mg respectively). After 24 h, the supernatant was aspirated and titrated via UV spectrophotometry (Agilent technologies, Cary 60 UV-Vis, Santa Clara, CA, USA) at 294 nm (absorption maximum). Optical densities were reported on a calibration curve established between 0 and 800 ppm of TA (correlation coefficient 0.9998). Drug adsorption was calculated as the difference between the initial and final concentrations of the TA solutions. The microparticles were conserved in the syringe until implant preparation.

### 2.4. Preparation of Particles/Plasma Composites and Subcutaneous Implantation

**Figure 1:**
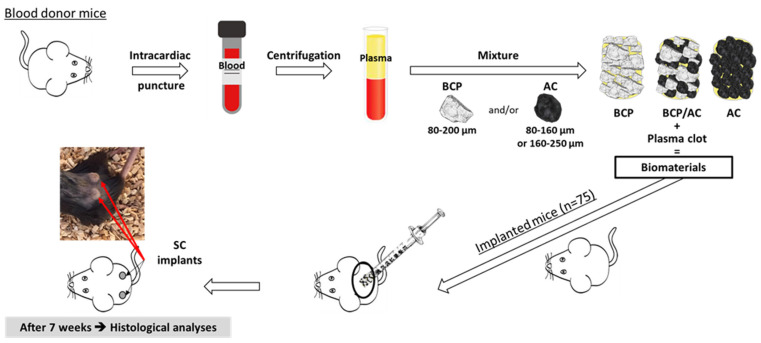
Experimental design of the study.

All animal procedures obtained the approval of the local animal health care committee (authorization reference numbers APAFIS#31398-2021042714514829 v3). The experimental design of the study is described in Figure 1. Whole blood was withdrawn on sodium citrate anticoagulant from anesthetized 10-week-old mice C57BL/6 mice (Janvier, Le Genest St. Isle, France) via intracardiac puncture. Plasma was obtained after blood centrifugation for 15 min at 3000× *g* at room temperature.

The different composite biomaterials were prepared in 1 mL syringes by adding 100 μL of plasma to the following preparations of calibrated particles, corresponding to a 50 µL final volume: (1) 50 mg of BCP (80–200 μm); (2) a mixture of 25 mg of BCP (80–200 µm) and 5 mg of AC (80–160 μm); (3) 10 mg of AC (80–160 μm); (4) 10 mg of AC_250_ (160–250 µm). When indicated, the drug (tacrolimus) was first adsorbed into the particles as described above. Plasma coagulation was triggered by adding 10 µL of a 2% CaCl_2_·2H_2_O solution. The syringes were left in an upright position to let the particles sediment and put at room temperature for 20 min to allow plasma clotting.

Subcutaneous implantation was performed on 10-week-old C57BL/6 mice anesthetized via the intraperitoneal injection of ketamine (90 mg/kg; Virbac, Carros, France) and xylasine (4.5 mg/kg; Ceva, Libourne, France). After cutting the syringe tip, the implants were pushed out in subcutaneous pockets beneath the dorsal skin. Each mouse received two implants. After 7 weeks, the animals were sacrificed with carbon dioxide and the implants were retrieved for histological analysis. For each condition (column 1) the exact number of mice implanted (column 2) and the exact number of implants analyzed (column 3) are shown in Table 1. In order to have sufficient data to apply statistics (see below) to our conditions, at least 7 implants were analyzed, corresponding to a minimum of 4 different animals.

### 2.5. Morphological Characterization of the Composites

Scanning electron microscopy (SEM, IT800SHL-JEOL, Croissy-sur-Seine, France) operating at 5 kV was used to investigate the morphology and the organization of the composite biomaterials before implantation. Particles embedded in a plasma clot were fixed overnight at 4 °C with a buffered 1.6% glutaraldehyde/0.1 M phosphate solution, rinsed, dehydrated in a graded ethanol series and dried at room temperature. They were then mounted on aluminum stubs with carbon tabs and sputter-coated with carbon before observation.

### 2.6. Histological Analyses

Histological analyses were performed after 7 weeks of SC implantation. The implants were fixed in 10% buffered formalin, then partially decalcified in 10% (*w*/*v*) EDTA solution (ICN Biomedical, Costa Mesa, CA, USA) for 48 h at room temperature and embedded in paraffin. Serial sections measuring 7 μm in thickness were prepared for hematoxylin and erythrosine staining (HE, Harris Hematoxylin solution, Sigma-Aldrich, Saint Quentin Fallavier, France; Eosin 0.5% alcoholic, Diapath, Voisins-le-Bretonneux, France).

Vectra Polaris (Akoya, Villebon-surYvette, France) was used at ×40 magnification to digitize the colored slides. For digital image analysis (DIA), a random forest-supervised classifier within the HALO platform (IndicaLab, Corrales, NM, USA) was applied as a machine learning method. DIA platforms were employed to assess active tissue, microparticles (BCP and/or AC), blood vessels and nucleated cells using various software tools, such as color deconvolution and cell segmentation algorithms (e.g., Watershed cell detection), along with supervised classifiers, as machine-learning methods. The initial step of analysis involved meticulous manual training of the machine learning classification to identify tissue patterns. Subsequently, tissue and cell segmentation were applied solely within the annotations designated by the machine learning classification. The analyses’ resolution was estimated to be below 1 μm.

The comparison between manual and automatic blood vessel counts is shown in the Supplementary Materials (Figure S1).

### 2.7. Porosity Analysis of AC and BCP Particles

The porous property of AC microparticles was determined via N_2_ adsorption/desorption isotherms at −196 °C in a volumetric analyzer (ASAP 2000, Micromeritics, Mérignac, France) after outgassing under vacuum at 80 °C for 12 h. The specific surface area calculated via the Brunauer–Emmett–Teller (BET) method, *S_BET_*, was obtained using the BET equation in the appropriate range of relative pressures [32]. The gas adsorption isotherms were used to evaluate the pore size distributions (PSDs) using the 2D-NLDFT-HS model for porous carbons with corrugated and chemically heterogenous surfaces [33].

### 2.8. Statistical Analysis

The statistical evaluation of data was performed using Student’s test. The experiment was repeated between seven and twelve times (Table 1), and the results presented are the mean of the values. Data are reported as ± standard deviation at a significance level of at * *p* < 0.05, ** *p* < 0.01 and *** *p* < 0.001.

## 3. Results

### 3.1. Comparison of Subcutaneous IMMATURE Bone Formation Induced by BCP, BCP/AC and AC Composites

**Figure 2:**
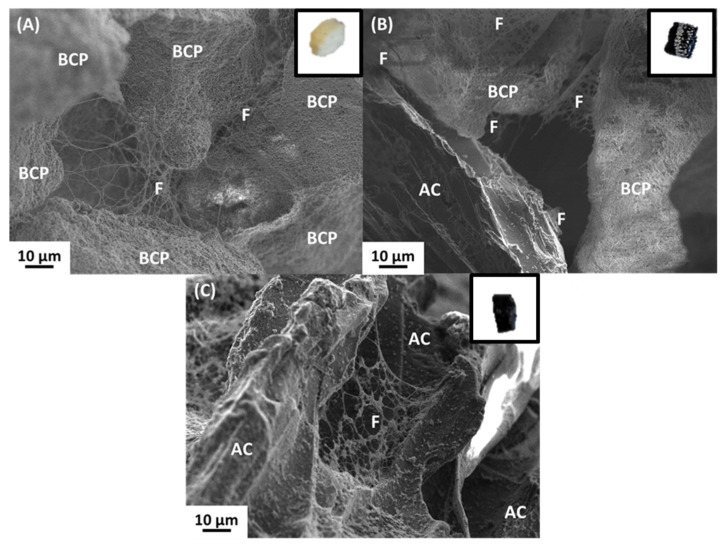
SEM images of (**A**) BCP particles 80–200 µm/plasma clot; (**B**) BCP/AC particles/plasma clot; and (**C**) AC particles 80–160 µm/plasma clot. The fibrin network is indicated (F). Inserts in A, B and C show a photograph of each biomaterial obtained after plasma coagulation.

We first tested the three following composites, constituting plasma clotted around (1) BCP particles (BCP/plasma), (2) AC particles (AC/plasma), or (3) a mixture of BCP and AC particles (BCP/AC/plasma), as described above. In all cases, plasma clotting around the microparticles resulted in the formation of a cohesive and malleable composite that could be easily handled (Figure 2, inserts). The microscopic organization of these biomaterials was analyzed using SEM as shown in Figure 2. This revealed the presence, for the three biomaterials, of a fibrin network linking the microparticles together, which confirmed that none of these materials prevented plasma coagulation.

**Figure 3:**
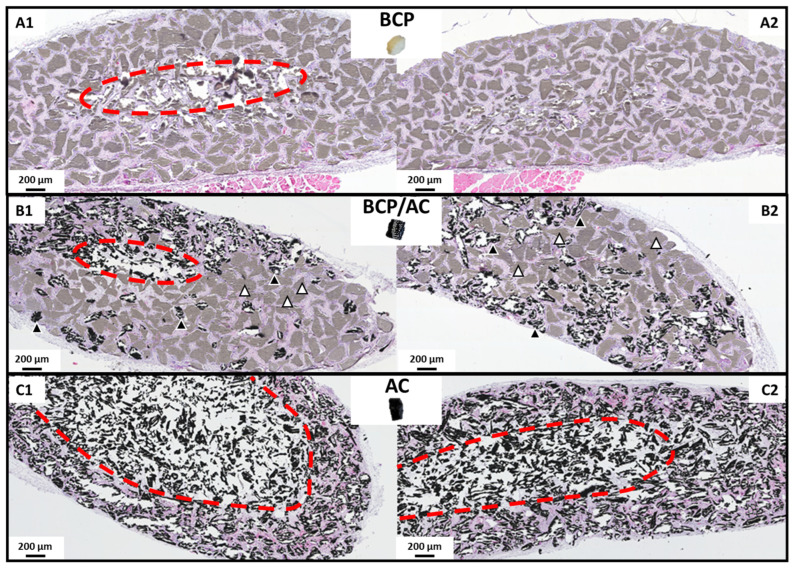
Light microscopy examination of the composite biomaterials: (**A1**,**A2**) BCP/plasma; (**B1**,**B2**) BCP/AC/plasma; and (**C1**,**C2**) AC/plasma. The images selected are representative of the worst (**left**) and best (**right**) results obtained for each type of biomaterial. In (**B1**,**B2**), the BCP microparticles appear in grey (white arrow) and the AC particles appear in black (black arrow). The red dotted line indicates the limit of tissue colonization.

**Figure 4:**
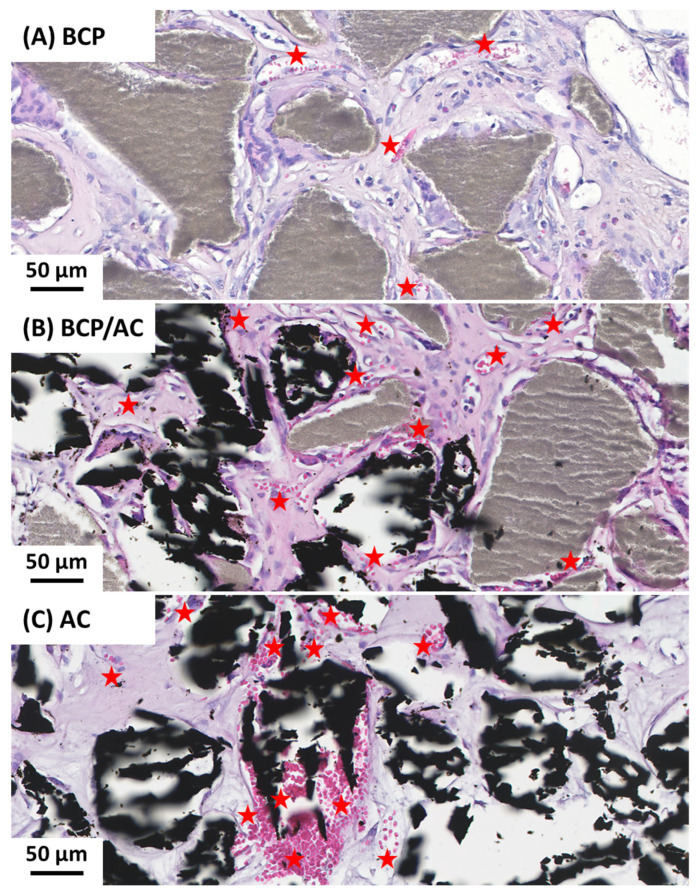
Light microscopy examination at a higher magnification of (**A**) BCP/plasma; (**B**) BCP/AC/plasma; and (**C**) AC/plasma. BCP microparticles are visible in grey, AC particles are visible in black and the blood vessels are visible in pink and indicated (red star).

**Figure 5:**
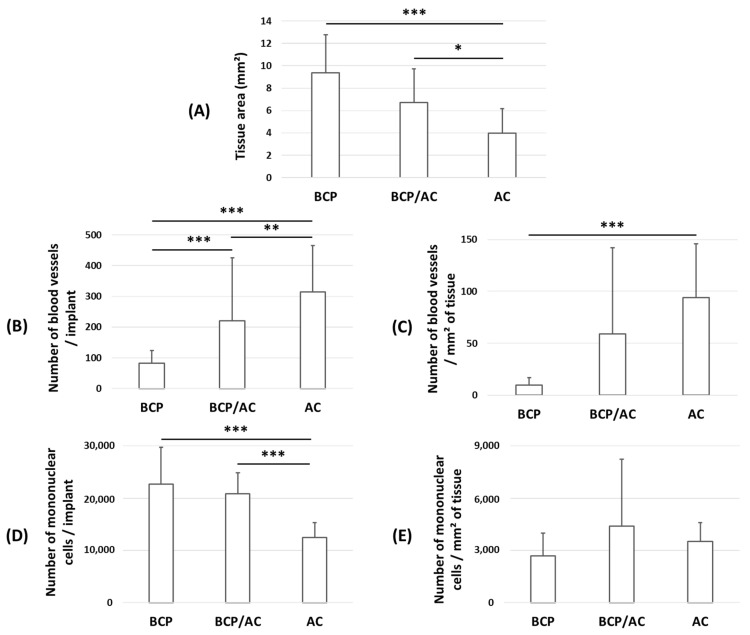
Quantification from histological sections of the three biomaterials described in Figure 3: (**A**) tissue area; (**B**) blood vessels enumerated either per implant or (**C**) per mm^2^ of tissue; (**D**) mononuclear cells per implant or (**E**) per mm^2^ of tissue. Mean ± standard deviation, * *p* < 0.05, ** *p* < 0.01 and *** *p* < 0.001.

Histological analysis performed after 7 weeks of SC implantation revealed (Figure 3, Figure 4 and Figure 5) significant differences between the three implant types. In all cases, we observed that the biomaterials were centripetally colonized by an immature collagenous tissue (named immature bone or woven bone, or bone-like tissue) constituted of a fibrillar bone matrix associated with a polymorphic cellular reaction with fibroblastic proliferation, many monocytes, granulocytes and macrophages. Multinucleated giant cells or osteoclast-like cells were observed surrounding the BCP and AC particles. Numerous vessels were distributed in all the implants. The limits of tissue colonization are highlighted by a red dotted line (Figure 3). As shown in Figure 3, the amount of newly formed tissue was similar in BCP implants and BCP/AC implants and much lower in AC implants. The quantification of tissue area (Figure 5A) confirmed these results and indicated that the differences between BCP and AC and between BCP/AC and AC were statistically significant. 

Observation at a higher magnification further revealed that the number and size of blood vessels were markedly higher in AC/plasma (Figure 4C) than in BCP/plasma implants (Figure 4A) and that these parameters were intermediate in BCP/AC implants (Figure 4B). This was confirmed after quantification (Figure 5B,C), whether the number of vessels was expressed per implant (Figure 5B) or per surface area of tissue (Figure 5C). Again, this number was intermediate in BCP/AC/plasma implants. Taken together, these results demonstrate that the addition of AC particles to BCP/plasma implants did not significantly modify the amount of tissue formation but was highly beneficial for tissue vascularization. In parallel, the number of mononuclear cells calculated per implant was reduced in the presence of AC particles (Figure 5D) but was similar in the three conditions when related to the surface area of collagenous tissue (Figure 5E).

**Figure 6:**
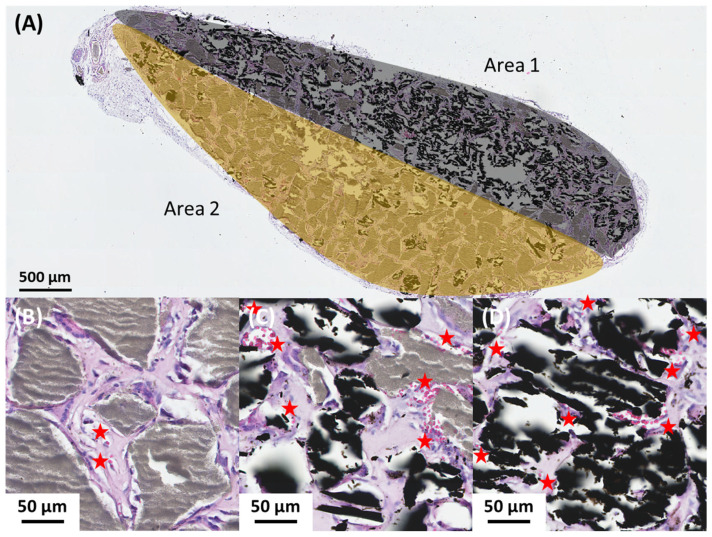
Light microscopy examination of a representative inhomogeneous BCP/AC/plasma implant. (**A**) Presentation of a whole implant with two areas: Area 1, carbon-rich area colored in grey, and Area 2, BCP-rich area colored in brown; higher magnification of (**B**) BCP-rich area; (**C**) BCP/AC area; and (**D**) AC-rich area. BCP microparticles are visible in grey, AC particles are visible in black and blood vessels are visible in pink and indicated (red star).

In the course of these experiments, we sometimes obtained BCP/AC/plasma implants in which the particle distribution was not homogeneous. As a result, some parts of the implants were more carbon-rich (Figure 6A, Area 1), while other parts were more BCP-rich (Figure 6A, Area 2). The histological analysis of the different areas of these implants allowed us to confirm the previous results. We observed that the areas concentrated with carbon particles were less well colonized but richer in blood vessels (Figure 6D). In parallel, in areas with a higher concentration of BCP particles, we observed better tissue colonization but a lower number of blood vessels (Figure 6B). In small areas where particle distribution was homogeneous, we found the same results as those described for BCP/AC/plasma implants; that is, similar colonization and an increase in the blood vessel number to those that observed with BCP alone (Figure 6C).

**Figure 7:**
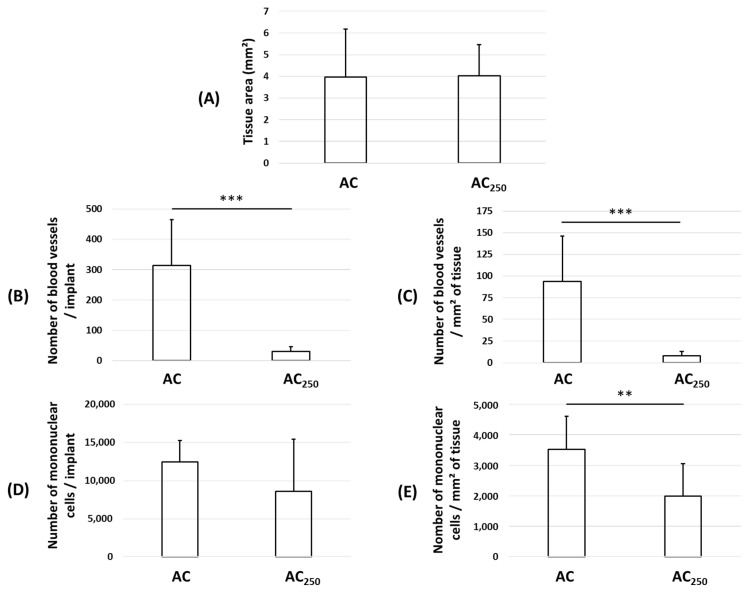
Quantification from histological sections of implants prepared with AC microparticles measuring 80–160 µm (AC) or 160–250 µm (AC_250_). Quantification of (**A**) tissue area; (**B**) blood vessel number per implant; (**C**) blood vessel number per mm^2^ of tissue; (**D**) mononuclear cell number per implant; and (**E**) mononuclear cell number per mm^2^ of tissue. Mean ± standard deviation; ** *p* < 0.01 and *** *p* < 0.001.

In another experiment, we asked whether or not the size of the carbon particles could have any effect on the parameters, that is, colonization and vascularization. We compared implants made of 80–160 µm AC particles with implants made of 160–250 µm AC particles (AC_250_). As shown by the quantification results in the Figure 7, similar tissue colonization was observed regardless of the size of the AC particles (Figure 7A). On the contrary, in the presence of the largest particles (AC_250_, 160–250 µm), the number of blood vessels, either calculated per implant or per unit of area, dropped dramatically (Figure 7B,C), as did, to a lower extent, the number of mononuclear cells (Figure 7D,E).

### 3.2. Influence of Biomaterial Impregnation with Solvents on Subcutaneous Immature Bone Formation

**Figure 8:**
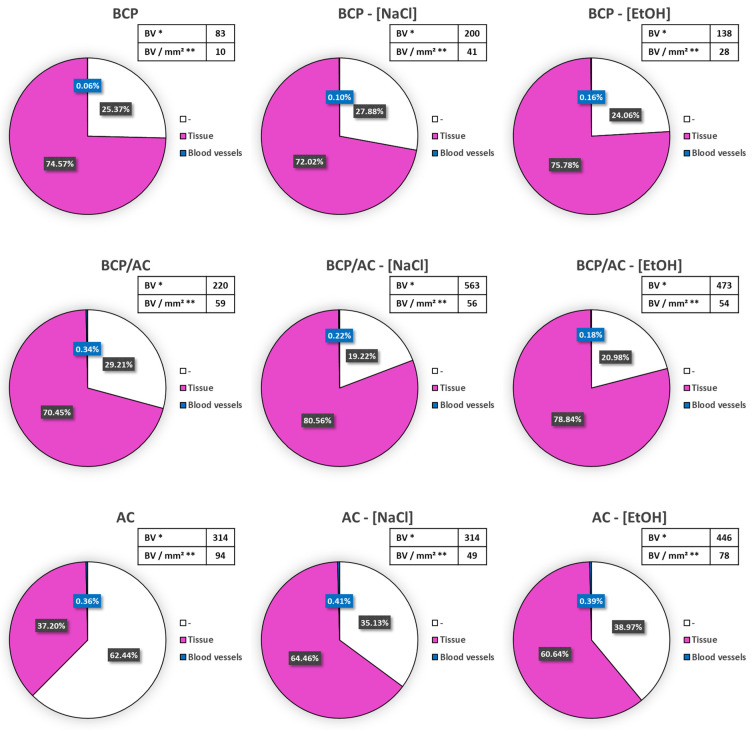
Representation of the relative proportion of immature bone tissue (pink colored) and blood vessels (blue colored) after quantification from histological sections. In the inserts, (*) the number of blood vessels (BV) per implant and (**) the number of blood vessels per mm^2^ of tissue (BV/mm^2^) are shown.

Before testing the effect of tacrolimus adsorption into the AC particles on ectopic bone-like tissue formation, we investigated whether adsorption with aqueous or alcoholic buffers could have an effect by itself on implant colonization and vascularization. AC and BCP particles were immersed either in isotonic NaCl solution (NaCl) or in 96% alcohol (EtOH), the latter being the solvent used to dissolve tacrolimus. After embedding in a plasma clot, these particles were implanted subcutaneously for 7 weeks following the same implantation protocol. The quantitative results (Figure 8, upper line) indicate that when BCP was immerged in NaCl or EtOH, tissue colonization (colored in pink) was similar in the three conditions, namely BCP (74.5%), BCP-[NaCl] (72.88%) and BCP-[EtOH] (75.78%). On the contrary, blood vessel number per mm^2^ of tissue increased in the presence of NaCl (41/mm^2^) and EtOH (28/mm^2^) compared with that in the presence of of dry BCP (10/mm^2^), indicating a beneficial effect of solvent adsorption on vascularization. When BCP/AC particles were immersed before implantation (Figure 8, second line), tissue colonization was increased (70.45% vs. 80.56% vs. 78.84%) and the total amount of blood vessels increased markedly in parallel, leading to an unchanged number of blood vessels per area of tissue (59 vs. 56 vs. 54 blood vessels per mm^2^). When AC particles were immersed before implantation (Figure 8, lower line), we observed a marked increase in tissue colonization (37.20 vs. 64.46 vs. 60.65%) together with the total number of blood vessels being unchanged, leading to a decrease in blood vessel density (94 vs. 49 vs. 78 vessels per mm^2^). Altogether, this experiment demonstrated that the NaCl or EtOH immersion of the particles, either AC, BCP or both, had a beneficial effect on immature bone tissue formation and vascularization after SC implantation (pictures taken from histological sections are shown in Figure S2). These conditions were used as controls in the experiments further conducted to test the effect of tacrolimus.

### 3.3. Particle Porosity and Tacrolimus (TA) Adsorption

As shown in the Table 2, BCP microparticles have a BET surface area close to 0 m^2^/g and AC microparticles exhibit a high BET surface area above 1500 m^2^/g. In the first case, the material is non-porous (BCP), and in the second, it is highly porous (AC) [34].

**Figure 9:**
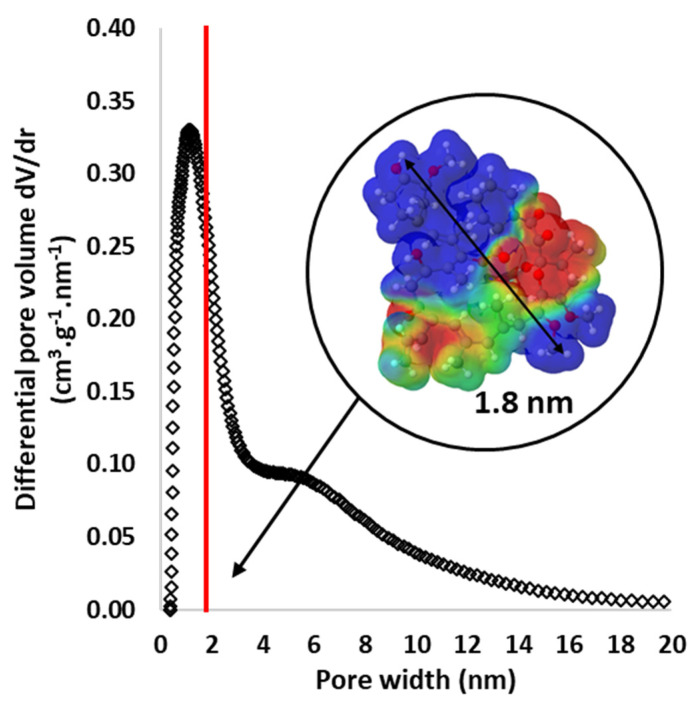
Gas adsorption data: pore size distribution of AC microparticles calculated via the application of the 2D-NLDFT model for carbon materials with corrugated surfaces. In the insert, the tridimensional representation of the tacrolimus (TA) molecule (C_44_H_69_NO_12_) using the Molview software is shown.

Secondly, as shown in the Figure 9, our experiments reveal that AC particles exhibit a narrow pore size distribution with a large volume of micropores (0.7 nm < pore size < 2 nm) and also some mesopores (2 nm < pore size < 50 nm). In parallel, the 3D representation of the tacrolimus (TA, C_44_H_69_NO_12_) molecule, visualized using the Molview software (https://molview.org/, Figure 9, insert), shows that TA is a very large molecule with a maximum length of 1.8 nm (as shown in the pore size distribution by a red vertical line). These representations demonstrate that the pore distribution of AC particles perfectly matches the adsorption of very large molecules such as tacrolimus or even larger molecules. Two concentrations of TA were tested, namely [TA_1_-EtOH] (800 ppm) and [TA_2_-EtOH] (160 ppm) solutions. TA adsorption by AC and BCP/AC particles was quantified by comparing these two initial concentrations (800 and 160 ppm) with their final concentrations, which were measured after particle immersion for 24 h. The results in Table 3 show the capacity and efficiency of AC particles to adsorb TA. 

### 3.4. Effect of Tacrolimus (TA) on Subcutaneous Bone-like Tissue Formation

**Figure 10:**
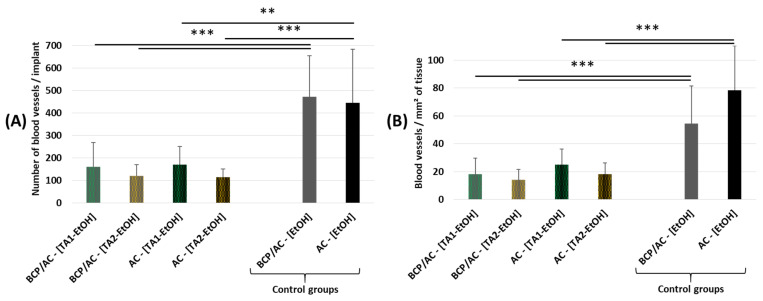
Quantification from histological sections of implants prepared with AC microparticles measuring 80-160 µm immersed either in tacrolimus solutions at two different concentrations or in EtOH (vehicle): quantification of blood vessel number (**A**) per implant and (**B**) per mm^2^ of bone -like tissue. Mean ± standard deviation; ** *p* < 0.01 and *** *p* < 0.001.

After immersion for 24 h either in the tacrolimus solutions (800 ppm or 160 ppm) or in EtOH solution alone (vehicle), the particles (see Table 3) were embedded in a plasma clot and implanted subcutaneously in mice as before. Histological analysis showed no effect of TA on the amount of immature bone tissue formed in the implants compared with that in the controls. On the contrary, TA adsorption into AC particles induced a dramatic and statistically significant decrease in the number of blood vessels, whether expressed per implant (Figure 10A) or per unit area of tissue area (Figure 10B). More precisely, for both types of implant, that is, BCP/AC-[TA-EtOH] or AC-[TA-EtOH], the number of blood vessels was divided by three in the presence of TA_1_ and divided by four in the presence of TA_2_.

## 4. Discussion

We have already described the bone-reconstructive properties of BRB, a biomaterial constituting calibrated BCP particles embedded in a plasma clot [9,31]. In recent experiments, we used activated carbon in the form of cloth and demonstrated that these tissues, when applied to bone defects in a rat femur, accelerated bone cortical repair and could be integrated in the newly formed bone [25,27]. Moreover, we also showed that AC cloth could be used as a drug delivery system [28]. 

We show here that AC can also be prepared in calibrated particle form, and the present study was conducted to investigate whether or not addition of AC to BCP microparticles could extend the properties of the BRB by allowing the in situ delivery of therapeutic agents. We thus tested, for the first time, the efficacy of AC particles and that of AC/BCP particles in combination, embedded in a plasma clot, in mice, in a model of SC bone-like tissue formation. This highly reproducible model has been used for several decades by our group and others to test the osteogenic potential of biomaterials [9,29,30,31].

Owing to our previous experiments with BRB (BCP/blood or plasma), which demonstrated that 80–200 µm calibrated BCP particles were optimal for bone reconstruction, AC particles were sieved to obtain a similar size range. Two batches of AC microparticles were thus prepared, and calibrated between 80 and 160 µm and between 160 and 250 µm. 

We then tested the feasibility of combining AC and BCP particles in the same biomaterial and the effect of this combination on SC immature bone formation. Our preliminary experiments revealed that the density of AC particles was five times lower than that of BCP, i.e., 50 mg of BCP and 10 mg of AC occupy the same 50 µL volume. Consequently, in order to have implants of an identical size, 50 µL, AC/plasma implants were formed with 10 mg of AC particles, BCP/AC/plasma implants constituted a combination of 25 mg of BCP and 5 mg of AC, and BCP/plasma were composed of 50 mg of BCP microparticles. First, we showed that AC particles alone in a plasma clot induce weak formation of ectopic bone compared with that observed with BCP particles. Unexpectedly, we observed that the number of blood vessels in these AC/plasma implants was very significantly greater than that in BCP/plasma implants. Moreover, not only was the number of vessels higher, but so was their average size. We observed the same proangiogenic effect of AC particles in BCP/AC/plasma to that of AC particles in BCP/plasma implants. In parallel, the number of nucleated cells remained high and stable in the newly formed tissue, suggesting a positive evolution of tissue formation. So far, the positive effect of AC on the number and the size of blood vessels is not understood. One possible explanation is that activated carbon, because of its adsorption capacity [35,36], captures molecules from the inner environment, such as growth factors or angiogenic molecules [37], which can be released in situ and stimulate angiogenesis. Further experiments will be necessary to deepen our understanding of this point. Nevertheless, this unexpected positive action of AC particles on implant vascularization, in the absence of any drug adsorption, is an extremely interesting beneficial effect since it is well known that the long-term survival of any type of graft is dependent on its neovascularization. It is likely that this potentiation of vascularization, which we observed here in an ectopic site by adding AC to BCP particles, will have an even greater positive impact on bone reconstruction following the implantation of this new BCP/AC/plasma biomaterial in bone defects. Future experiments will be necessary to assess this beneficial effect in bony sites as well as to assess the optimal ratio of BCP/AC microparticles. Nevertheless, our results demonstrate that a small amount of AC microparticles is sufficient to significantly increase the neovascularization of the ectopic tissues.

One experiment that we carried out demonstrated that the implants made from the largest particles embedded in coagulated plasma, that is, particles measuring 160–250 µm, produced very loose collagenous tissue and a significantly lower number of blood vessels than those in implants made from the smallest particles. This explains why all the other experiments were designed with AC particles measuring 80–160 µm, BCP particles measuring 80–200 µm, or a combination of the two. 

Our study further aimed to determine the feasibility of using the adsorption capacity of AC to deliver a drug into implants. Tacrolimus (TA) was an interesting drug with which to test the adsorption capacity of the AC microparticles because of its relatively large size (803.5 Da) and low solubility (4–12 μg/mL in water) [38]. We demonstrate here that the porosity of AC particles, which combines micropores and mesopores, enables the adsorption of this large molecule. TA was also chosen since it is an immunosuppressive agent that others groups have used in previous studies for its ability to influence bone formation in various models [39]. Among these studies, some authors describe that TA treatment increased bone formation markers in patients with rheumatoid arthritis [40]. Others have shown that, in rats, TA increases bone resorption without affecting bone formation, leading to bone loss [41]. Nabavi et al. described a model where TA (10 μg/mL, 100 μg/mL, and 1000 μg/mL) was loaded into collagen type I hydrogel, which was then surrounded by film made of gelatin and polycaprolactone and placed in the calvarias defect of Wistar rats. They demonstrated that this hydrogel could promote the repair of cranial bone defects in rats [42]. In line with a piece of previous work, we chose TA because we found that the adaptive immune response inhibits ectopic mature bone formation induced by bone marrow stromal cells/BCP/plasma implants in immune-competent mice [31]. We thus hypothesized that immune suppression brought about in situ by TA via AC particles could have a beneficial effect on SC bone-like tissue formation. Since TA is soluble in ethanol, EtOH and NaCl adsorbed on AC were used as controls. The results obtained in these control conditions demonstrate that the adsorption of EtOH or NaCl into AC particles not only has no deleterious effect but exerts a slightly positive effect through an increase in blood vessel number when compared with dry particles. Hypotheses can be made to explain this unexpected positive impact of using an implant made of “wet” instead of “dried” particles. The immersion of BCP particles in ethanol or NaCl for 24 h may induce surface modifications such as the partial dissolution of the surface layer. This modified surface could then exhibit enhanced biocompatibility/osteointegration properties, favoring new vessel formation, for example. In the case of AC particles, the presence of solvent trapped in their micropores could modify the kinetics of exchange with the inner medium, which may promote biological processes including immature bone matrix formation. It is therefore conceivable that the immersion of BCP/AC particles in a solvent prior to implantation, carried out by modulating the adsorption of the inner medium and creating surface modifications, promotes biological processes including immature bone matrix formation and the growth of new vessels. Further studies will be necessary to understand the mechanisms involved in the effects of solvents on BCP and AC particles and their influence on immature bone tissue formation and vascularization.

The adsorption of TA-[EtOH] into AC particles after immersion for 24 h in two different concentrations resulted in a two-load range. Our results revealed that BCP/AC-[TA-EtOH] and AC-[TA-EtOH] particles embedded in plasma induced the formation of immature bone tissue with a dramatic drop in blood vessel numbers when compared with those in their respective controls. This effect was similar for AC and BCP/AC implants and for the two loads of TA, with a reduction in the number of blood vessels by a factor of 3 or 4. Note that the deleterious effect of TA is probably limited by the beneficial effect of ethanol on the number of blood vessels. Further experiments are needed to explain this negative effect of TA on angiogenesis, but this deleterious effect of TA on implant vascularization allowed us to demonstrate that TA adsorbed into AC particles was indeed released in situ into the implants. This result provides the proof of concept that AC particles can carry molecules of interest to implanted sites. 

Taken together, our results indicate that the addition of AC to BCP particles in this model not only results in better-vascularized ectopic bone tissue, but also allows the delivery of therapeutic molecules to the implant site, including large ones such as TA molecules. This strongly suggests that different types of molecules could be delivered by these particles and, consequently, that different therapeutic effects could therefore possibly be obtained. This important property of AC is complementary to the osteogenic effect of BCP, which has no adsorption properties, as confirmed here. 

In clinical practice, the reconstruction of a bone defect may be associated with co-morbidities such as infection or neoplastic disease. The use of biomaterials that are not only effective for bone reconstruction, but that can also deliver drugs such as an anti-infectious or anti-neoplastic drugs in situ, is a major step forward. Our first results provide a proof of concept that activated carbon particles can be used to adsorb drugs, including large ones, which are released in situ into the site to be reconstructed. This feasibility study should be extended to implantation in bony sites and larger animals, using anti-infectious or anti-neoplastic drugs in adapted models.

## Figures and Tables

**Table 1:** Composition and number of implants. For each condition (column 1), the exact number of mice implanted (column 2) and the exact number of implants analyzed (column 3) are indicated. Tacrolimus was used at 2 concentrations namely, TA_1_ and TA_2_.

Particle–[Drug–Solvent] Associated with Plasma	*n* (Mice)	*n* (Implants Analyzed)
BCP	5	9/10
BCP/AC	6	12/12
AC	5	10/10
AC_250_	6	8/12
BCP-[NaCl]	5	10/10
BCP-[EtOH]	5	10/10
BCP/AC-[NaCl]	5	9/10
BCP/AC-[EtOH]	5	8/10
AC-[NaCl]	5	10/10
AC-[EtOH]	5	7/10
BCP/AC-[TA_1_-EtOH]	6	11/12
BCP/AC-[TA_2_-EtOH]	6	12/12
AC-[TA_1_-EtOH]	6	11/12
AC-[TA_2_-EtOH]	5	9/10

**Table 2:** BET surface area value quantified for both types of particles.

	S_BET_ (m^2^/g)
BCP	2
AC ^1^	1562

^1^ AC sieved to 80–160 µm.

**Table 3:** Quantification of TA adsorbed into BCP (25 mg)/AC (5 mg) and AC (10 mg) microparticles after immersion for 24 h in TA_1_ (800 ppm) or TA_2_ (160 ppm) solution at room temperature. Adsorption is presented either as the total mass of TA adsorbed (µg_TA_) or the mass of TA adsorbed per g of particles (mg_TA_/g_AC_).

		BCP/AC—[TA_1_-EtOH] (800 ppm)	BCP/AC—[TA_2_-EtOH] (160 ppm)	AC—[TA_1_-EtOH] (800 ppm)	AC—[TA_2_-EtOH] (160 ppm)
m_TA_ (µg)	Mean ± SD	95 ± 10	30 ± 4	120 ± 10	45 ± 8
Q_TA_ (mg/g)	Mean ± SD	19 ± 2	6 ± 1	24 ± 2	9 ± 2

## Data Availability

Data are contained within the article and Supplementary Materials.

## Supplementary Materials

The following supporting information can be downloaded at https://www.mdpi.com/article/10.3390/ma17081749/s1: Figure S1: Quantification of blood vessel number from histological sections either via manual counting (grey histogram) or via automatic counting (white histogram) for the three biomaterials; Figure S2: Light microscopy pictures of (A) BCP/AC-[NaCl]; (B) BCP/AC-[EtOH]; (C) AC-[NaCl] and (D) AC-[EtOH] composites.

## References

[1] Aggarwal D., Kumar V., Sharma S. (2022). Drug-Loaded Biomaterials for Orthopedic Applications: A Review. J. Control. Release.

[2] VK A.D., Ray S., Arora U., Mitra S., Sionkowska A., Jaiswal A.K. (2022). Dual Drug Delivery Platforms for Bone Tissue Engineering. Front. Bioeng. Biotechnol..

[3] Fernandez De Grado G., Keller L., Idoux-Gillet Y., Wagner Q., Musset A.-M., Benkirane-Jessel N., Bornert F., Offner D. (2018). Bone Substitutes: A Review of Their Characteristics, Clinical Use, and Perspectives for Large Bone Defects Management. J. Tissue Eng..

[4] Bouler J.M., Pilet P., Gauthier O., Verron E. (2017). Biphasic Calcium Phosphate Ceramics for Bone Reconstruction: A Review of Biological Response. Acta Biomater..

[5] Chu K.-T., Ou S.-F., Chen S.-Y., Chiou S.-Y., Chou H.-H., Ou K.-L. (2013). Research of Phase Transformation Induced Biodegradable Properties on Hydroxyapatite and Tricalcium Phosphate Based Bioceramic. Ceram. Int..

[6] Houmard M., Fu Q., Genet M., Saiz E., Tomsia A.P. (2013). On the Structural, Mechanical, and Biodegradation Properties of HA/β-TCP Robocast Scaffolds: Bone-Substitute Material in Various Tissue-Engineering Applications. J. Biomed. Mater. Res..

[7] Miranda P., Pajares A., Saiz E., Tomsia A.P., Guiberteau F. (2008). Mechanical Properties of Calcium Phosphate Scaffolds Fabricated by Robocasting. J. Biomed. Mater. Res..

[8] Daculsi G., Passuti N., Martin S., Deudon C., Legeros R.Z., Raher S. (1990). Macroporous Calcium Phosphate Ceramic for Long Bone Surgery in Humans and Dogs. Clinical and Histological Study. J. Biomed. Mater. Res..

[9] Balaguer T., Boukhechba F., Clavé A., Bouvet-Gerbettaz S., Trojani C., Michiels J.-F., Laugier J.-P., Bouler J.-M., Carle G.F., Scimeca J.-C. (2010). Biphasic Calcium Phosphate Microparticles for Bone Formation: Benefits of Combination with Blood Clot. Tissue Eng. Part A.

[10] Balaguer T., Fellah B.H., Boukhechba F., Traverson M., Mouska X., Ambrosetti D., Dadone B., Michiels J., Amri E., Trojani C. (2018). Combination of Blood and Biphasic Calcium Phosphate Microparticles for the Reconstruction of Large Bone Defects in Dog: A Pilot Study. J. Biomed. Mater. Res..

[11] Ahmed M.J. (2017). Adsorption of Non-Steroidal Anti-Inflammatory Drugs from Aqueous Solution Using Activated Carbons: Review. J. Environ. Manag..

[12] Mansour F., Al-Hindi M., Yahfoufi R., Ayoub G.M., Ahmad M.N. (2018). The Use of Activated Carbon for the Removal of Pharmaceuticals from Aqueous Solutions: A Review. Rev. Environ. Sci. Biotechnol..

[13] Do D.D., Junpirom S., Do H.D. (2009). A New Adsorption–Desorption Model for Water Adsorption in Activated Carbon. Carbon.

[14] Aschermann G., Schröder C., Zietzschmann F., Jekel M. (2019). Organic Micropollutant Desorption in Various Water Matrices-Activated Carbon Pore Characteristics Determine the Reversibility of Adsorption. Chemosphere.

[15] Lopez De Armentia S., Del Real J.C., Paz E., Dunne N. (2020). Advances in Biodegradable 3D Printed Scaffolds with Carbon-Based Nanomaterials for Bone Regeneration. Materials.

[16] Wei C., Jin X., Wu C., Zhang W. (2022). Injectable Composite Hydrogel Based on Carbon Particles for Photothermal Therapy of Bone Tumor and Bone Regeneration. J. Mater. Sci. Technol..

[17] Babuska V., Kasi P.B., Chocholata P., Wiesnerova L., Dvorakova J., Vrzakova R., Nekleionova A., Landsmann L., Kulda V. (2022). Nanomaterials in Bone Regeneration. Appl. Sci..

[18] Rejman J., Oberle V., Zuhorn I.S., Hoekstra D. (2004). Size-Dependent Internalization of Particles via the Pathways of Clathrin- and Caveolae-Mediated Endocytosis. Biochem. J..

[19] Yang G., Phua S.Z.F., Bindra A.K., Zhao Y. (2019). Degradability and Clearance of Inorganic Nanoparticles for Biomedical Applications. Adv. Mater..

[20] Wu C., Chen H., Wu X., Cong X., Wang L., Wang Y., Yang Y., Li W., Sun T. (2017). The Influence of Tumor-Induced Immune Dysfunction on the Immune Cell Distribution of Gold Nanoparticles In Vivo. Biomater. Sci..

[21] Aoki K., Usui Y., Narita N., Ogiwara N., Iashigaki N., Nakamura K., Kato H., Sano K., Ogiwara N., Kametani K. (2009). A Thin Carbon-Fiber Web as a Scaffold for Bone-Tissue Regeneration. Small.

[22] Boehm A., Meininger S., Tesch A., Gbureck U., Müller F. (2018). The Mechanical Properties of Biocompatible Apatite Bone Cement Reinforced with Chemically Activated Carbon Fibers. Materials.

[23] Chudoba D., Łudzik K., Jażdżewska M. (2022). Carbon Fibres as Potential Bone Implants with Controlled Doxorubicin Release. Sci. Rep..

[24] Zhao X., Yang Z., Liu Q., Yang P., Wang P., Wei S., Liu A., Zhao Z. (2022). Potential Load-Bearing Bone Substitution Material: Carbon-Fiber-Reinforced Magnesium-Doped Hydroxyapatite Composites with Excellent Mechanical Performance and Tailored Biological Properties. ACS Biomater. Sci. Eng..

[25] Olivier F., Sarou-Kanian V., Fayon F., Bonnamy S., Rochet N. (2022). In Vivo Effectiveness of Carbonated Calcium-deficient Hydroxyapatite-coated Activated Carbon Fiber Cloth on Bone Regeneration. J. Biomed. Mater. Res..

[26] Mi L., Li F., Xu D., Liu J., Li J., Zhong L., Liu Y., Bai N. (2024). Performance of 3D Printed Porous Polyetheretherketone Composite Scaffolds Combined with Nano-Hydroxyapatite/Carbon Fiber in Bone Tissue Engineering: A Biological Evaluation. Front. Bioeng. Biotechnol..

[27] Olivier F., Drouet C., Marsan O., Sarou-Kanian V., Rekima S., Gautier N., Fayon F., Bonnamy S., Rochet N. (2023). Long-Term Fate and Efficacy of a Biomimetic (Sr)-Apatite-Coated Carbon Patch Used for Bone Reconstruction. J. Funct. Biomater..

[28] Olivier F., Bonnamy S., Rochet N., Drouet C. (2021). Activated Carbon Fiber Cloth/Biomimetic Apatite: A Dual Drug Delivery System. Int. J. Mol. Sci..

[29] Kuznetsov S.A., Krebsbach P.H., Satomura K., Kerr J., Riminucci M., Benayahu D., Robey P.G. (1997). Single-Colony Derived Strains of Human Marrow Stromal Fibroblasts Form Bone after Transplantation In Vivo. J. Bone Miner. Res..

[30] Boukhechba F., Balaguer T., Bouvet-Gerbettaz S., Michiels J.-F., Bouler J.-M., Carle G.F., Scimeca J.-C., Rochet N. (2011). Fate of Bone Marrow Stromal Cells in a Syngenic Model of Bone Formation. Tissue Eng. Part A.

[31] Bouvet-Gerbettaz S., Boukhechba F., Balaguer T., Schmid-Antomarchi H., Michiels J.-F., Scimeca J.-C., Rochet N. (2014). Adaptive Immune Response Inhibits Ectopic Mature Bone Formation Induced by BMSCs/BCP/Plasma Composite in Immune-Competent Mice. Tissue Eng. Part A.

[32] Brunauer S., Emmett P.H., Teller E. (1938). Adsorption of Gases in Multimolecular Layers. J. Am. Chem. Soc..

[33] Jagiello J., Olivier J.P. (2013). 2D-NLDFT Adsorption Models for Carbon Slit-Shaped Pores with Surface Energetical Heterogeneity and Geometrical Corrugation. Carbon.

[34] Heidarinejad Z., Dehghani M.H., Heidari M., Javedan G., Ali I., Sillanpää M. (2020). Methods for Preparation and Activation of Activated Carbon: A Review. Environ. Chem. Lett..

[35] Mestre A.S., Pires J., Nogueira J.M.F., Parra J.B., Carvalho A.P., Ania C.O. (2009). Waste-Derived Activated Carbons for Removal of Ibuprofen from Solution: Role of Surface Chemistry and Pore Structure. Bioresour. Technol..

[36] Jeirani Z., Niu C.H., Soltan J. (2017). Adsorption of Emerging Pollutants on Activated Carbon. Rev. Chem. Eng..

[37] Inoue S., Kiriyama K., Hatanaka Y., Kanoh H. (2015). Adsorption Properties of an Activated Carbon for 18 Cytokines and HMGB1 from Inflammatory Model Plasma. Colloids Surf. B Biointerfaces.

[38] Tamura S., Ohike A., Ibuki R., Amidon G.L., Yamashita S. (2002). Tacrolimus Is a Class II Low-Solubility High-Permeability Drug: The Effect of P-Glycoprotein Efflux on Regional Permeability of Tacrolimus in Rats. J. Pharm. Sci..

[39] Ding Q., Zhang S., Liu X., Zhao Y., Yang J., Chai G., Wang N., Ma S., Liu W., Ding C. (2023). Hydrogel Tissue Bioengineered Scaffolds in Bone Repair: A Review. Molecules.

[40] Kang K.Y., Ju J.H., Song Y.W., Yoo D.-H., Kim H.-Y., Park S.-H. (2013). Tacrolimus Treatment Increases Bone Formation in Patients with Rheumatoid Arthritis. Rheumatol. Int..

[41] Kanda J., Izumo N., Furukawa M., Shimakura T., Yamamoto N., Takahashi H.E., Asakura T., Wakabayashi H. (2018). Effects of the Calcineurin Inhibitors Cyclosporine and Tacrolimus on Bone Metabolism in Rats. Biomed. Res..

[42] Nabavi M.H., Salehi M., Ehterami A., Bastami F., Semyari H., Tehranchi M., Nabavi M.A., Semyari H. (2020). A Collagen-Based Hydrogel Containing Tacrolimus for Bone Tissue Engineering. Drug Deliv. Transl. Res..

