# Supplementary material for: Biphasic Calcium Phosphate and Activated Carbon Microparticles in a Plasma Clot for Bone Reconstruction and In Situ Drug Delivery: A Feasibility Study
Source: Materials (Basel). 2024 Apr 11;17(8):1749. doi: 10.3390/ma17081749 (PMC11051311; doi:10.3390/ma17081749)
Supplement: Supplementary file 1 [file materials-17-01749-s001.zip › materials-2895406-supplementary.pdf]

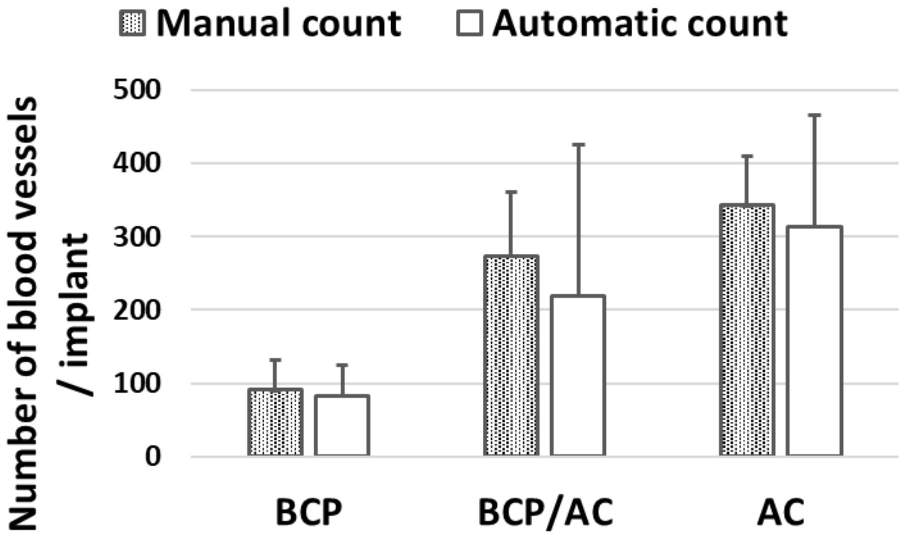


**Figure S1.** Quantification of blood vessel number from histological sections either by manual counting (grey histogram) or by automatic counting (white histogram) for the three biomaterials.

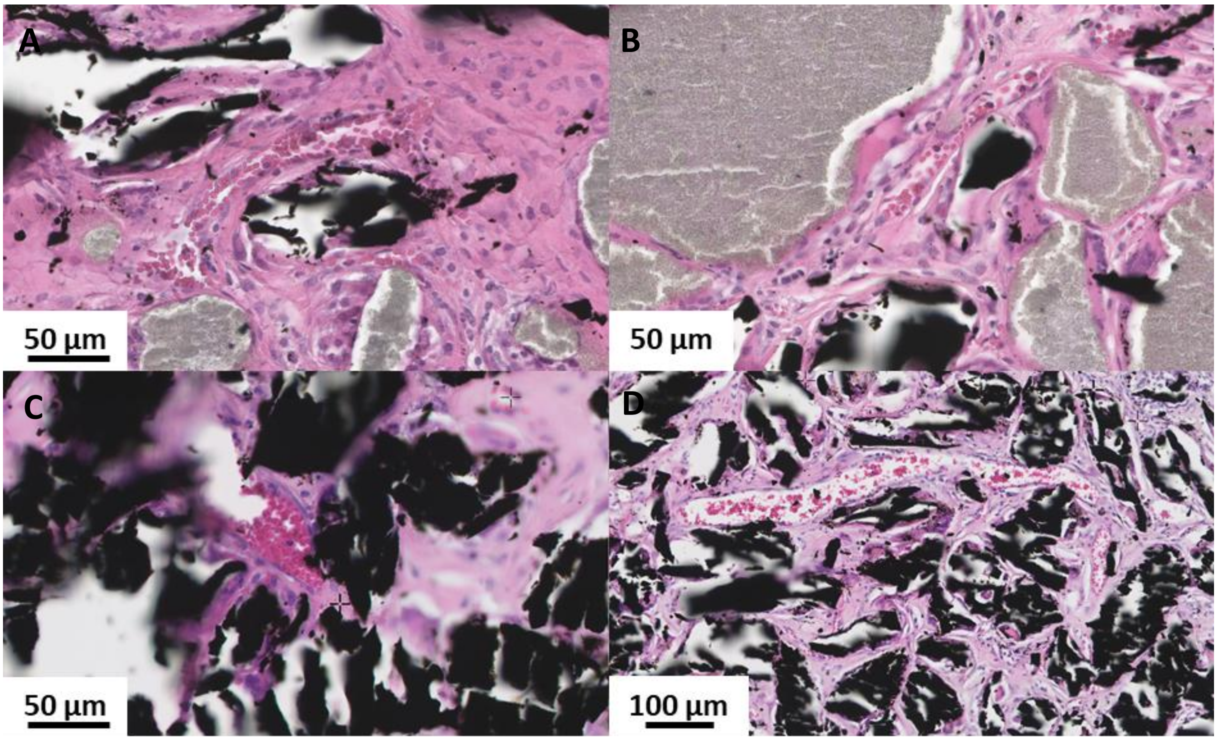


**Figure S2.** Light microscopy pictures of: (A) BCP/AC - [NaCl]; (B) BCP/AC - [EtOH]; (C) AC - [NaCl] and (D) AC - [EtOH] composites.
